# Draft Genome Sequences of the Antimicrobial Producers *Pseudomonas* sp. TAA207 and *Pseudomonas* sp. TAD18 Isolated from Antarctic Sediments

**DOI:** 10.1128/genomeA.00728-16

**Published:** 2016-07-28

**Authors:** Luana Presta, Ilaria Inzucchi, Emanuele Bosi, Marco Fondi, Elena Perrin, Isabel Maida, Elisangela Miceli, Maria Luisa Tutino, Angelina Lo Giudice, Donatella de Pascale, Renato Fani

**Affiliations:** a Department of Biology, University of Florence, Florence, Italy; b Institute of Protein Biochemistry, National Research Council, Naples, Italy; c Department of Chemical Sciences, University of Naples Federico II, Naples, Italy; d Institute for the Coastal Marine Environment, National Research Council (IAMC-CNR), Messina, Italy; e Department of Biological and Environmental Sciences, University of Messina, Messina, Italy

## Abstract

We report here the draft genome sequence of the *Pseudomonas* sp. TAA207 and *Pseudomonas* sp. TAD18 strains, isolated from Antarctic sediments during a summer campaign near coastal areas of Terra Nova Bay (Antarctica). Genome sequence knowledge allowed the identification of genes associated with the production of bioactive compounds and antibiotic resistance. Furthermore, it will be instrumental for comparative genomics and the fulfillment of both basic and application-oriented investigations.

## GENOME ANNOUNCEMENT

Antarctica provides one of the largest unexplored sources of biodiversity. Here, the continuous environmental challenges led to extremely adapted living forms that may be sources of potentially novel, untapped gene functions. Particularly, it has been shown how microorganisms are claimed to be a reservoir of biotechnologically relevant molecules, such as antibiotics (1–6).

Here, we report the genome sequences of two *Pseudomonas* sp. strains, TAA207 and TAD18, isolated from Antarctic sediments during a summer campaign near the coastal areas of Terra Nova Bay (Antarctica). These bacteria have been screened for antimicrobial activity against human pathogens. The results obtained show how they totally inhibited 40 strains belonging to the *Burkholderia cepacia* complex (BCC), most of which are affiliated to the species *Burkholderia cenocepacia* and *Burkholderia multivorans*, two of the most important pathogens in immunocompromised patients affected by cystic fibrosis disease. Also, they produce antibiofilm molecules acting against *Staphylococcus aureus* and *Pseudomonas aeruginosa* (7).

Both genome sequences of *Pseudomonas* sp. TAA207 and *Pseudomonas* sp. TAD18 were determined through a paired-end approach using the Illumina/Solexa genome analyzer IIx platform at the Institute of Applied Genomics and IGA Technology Services Srl (University of Udine, Italy). A total of 11,007,120 and of 12,698,315 reads were obtained for *Pseudomonas* sp. TAA207 and *Pseudomonas* sp. TAD18, respectively. Low-quality sequences were trimmed with StreamingTrim (8), and the remaining were assembled with SPAdes genome assembler version 3.6.1 (9). Only contigs longer than 1,000 bp were embedded in the final version of the draft genomes, which are 4,900,197-bp long for *Pseudomonas* sp. TAA207 (72 contigs, 453× average coverage, 57% GC content) and 4,917,586-bp long for *Pseudomonas* sp. TAD18 (82 contigs, average coverage 521×, 57.24% GC content).

Annotation was then performed using Prokka (10), which identified 4,379 and 4,403 genes for *Pseudomonas* sp. TAA207 and *Pseudomonas* sp. TAD18, respectively. Among these, 4,028 are protein-encoding genes in the former organism, and 4,220 are in the latter one.

The genome sequences allowed comparative genomics analysis to check for the presence of genetic traits involved in secondary metabolite biosynthesis. The analysis was performed within the antiSMASH shell (11), revealing that both genomes harbor gene clusters encoding molecules involved in inhibitory activities. Particularly, the two strains embed gene clusters similar to those coding for aryl-polyene, terpene, bacteriocin, and nonribosomal peptide synthase. Additionally, *Pseudomonas* sp. TAA207 contains a cluster involved in microcin production.

Further, we investigated the possibility that both strains possess antibiotic resistance genes in their pool by probing their sequences in the Comprehensive Antibiotic Resistance Database (CARD). The outcome yields strong indications that both genomes have genes coding for general efflux pumps, alongside several genes conferring resistance to specific classes of antibiotics, including chloramphenicol, fluoroquinilone, beta-lactam, trimethoprim, tetracycline, polymyxin, aminoglycoside, and rifampin.

### Nucleotide sequence accession numbers.

The whole-genome shotgun projects of *Pseudomonas* sp. TAA207 and *Pseudomonas* sp. TAD18 have been deposited at GenBank under the accession numbers LLWJ00000000 and LLWI00000000, respectively. The versions described in this paper are the first versions, LLWJ01000000 and LLWI01000000.
